# Voices of Plurality: Linguistic Diversity and Social Interactions in Ugandan Polygamous Marriages

**DOI:** 10.12688/f1000research.163648.2

**Published:** 2025-08-04

**Authors:** Elizabeth Kyomugisha

**Affiliations:** 1 College of Humanities and Social Sciences, Kampala International University, Kampala, Central Region, Uganda

**Keywords:** multilingualism, polygamy, Uganda, language and identity.

## Abstract

**Background:**

Polygamy remains a significant marital institution in Uganda, where multilingualism intersects with family structure, gender roles, and social status. While legal and health implications of polygamous unions have been studied, little attention has been paid to the role of language in shaping intra-household dynamics. This study investigates how linguistic diversity within Ugandan polygamous marriages reflects and constructs social hierarchies, emotional relationships, and marital strategies.

**Methods:**

This qualitative research analyzes 200 anonymized micro-narratives collected between 2023 and 2024 via the SIDINL Newsletters platform, spanning 20 Ugandan languages. Narrative analysis, sociolinguistic analysis, and discourse analysis were employed to examine language use in polygamous households. Stories were gathered from husbands, co-wives, children, and in-laws, transcribed, translated, and coded for themes such as hierarchy, emotional tone, and language switching.

**Results:**

Linguistic choices, such as honorifics, metaphors, and strategic code-switching, consistently signaled status, emotional positioning, and power dynamics within polygamous families. Multilingualism was used not only to navigate household conflict and intimacy but also as a tool for forming polygamous unions, particularly in urban, border, or migratory contexts. Regional storytelling patterns revealed culturally specific framings of polygamy: moral and spiritual in the West, inheritance-based in the North, pragmatic in the East, and justice-oriented in the West Nile. Selective multilingualism emerged as a strategy where language fluency influenced marriage decisions and access to social capital.

**Conclusions:**

Language functions as both a social instrument and a strategic resource in Ugandan polygamous marriages. It actively shapes family hierarchies, emotional dynamics, and mobility. Recognizing the role of language in household organization is essential for understanding marital structures in multilingual societies. These findings highlight the need for culturally grounded, linguistically sensitive approaches in both research and policy design.

## Introduction

### Background

Polygamous marriage remains a significant social institution in many parts of Uganda, particularly among communities where lineage, land ownership, and fertility are deeply tied to cultural identity ( Amone, 2020; Fenske, 2015). Though polygamy is often debated in public policy and human rights discourse, it continues to serve complex social and economic functions, offering, in some contexts, a means of consolidating wealth, strengthening kinship ties, or maintaining communal land rights. However, as polygamy intersects with gender, power, and modernization, new questions arise about how individuals within these marriages navigate relationships, roles, and resources.

At the same time, Uganda’s multilingual landscape adds another layer of complexity to family and social structures. With over 40 indigenous languages and multiple regional lingua francas, Ugandan society is deeply shaped by language diversity ( Nakayiza, 2016). Language plays a central role in everyday interactions, and studies have shown that it can influence social cooperation, identity, and perceptions of power ( Clist and Verschoor, 2017). In the context of family life, multilingualism often intersects with broader social factors, such as migration, education, and economic access, shaping how authority is expressed and negotiated within households ( Namazzi and Kendrick, 2014; Reh, 2004).

While existing literature has explored the legal, health, and gender implications of polygamy ( Ahinkorah, 2021; Johnson-Peretz et al., 2024), little attention has been paid to the linguistic dimensions of polygamous marriage. However, language is not merely a communicative tool in these households, it is a vehicle for social differentiation, emotional connection, and strategic navigation of everyday life. From the use of honorifics and metaphor to deliberate code-switching and selective multilingualism, language reveals much about the inner dynamics of cohabitation, hierarchy, and emotional power within polygamous unions. This dimension is especially underexplored in Uganda, where linguistic practices vary widely across regions and cultural groups ( Lorenz, 2019; Mukama, 1998).

### Research questions

This study seeks to fill that gap by examining how language both reflects and shapes social roles within polygamous marriages in Uganda. Drawing on a rich dataset of 200 narratives collected through the SIDINL Newsletter micro-storytelling platform between 2023 and 2024, this research investigates how individuals in polygamous and multilingual households use language to assert status, manage conflict, express intimacy, and navigate identity. The narratives span 20 different Ugandan languages, offering a rare opportunity to analyze how cultural variation is embedded in linguistic expression.

The central research questions guiding this study are: How does language use within polygamous marriages reflect and negotiate household hierarchy, emotional relationships, and social roles? How do regional and linguistic differences shape the way polygamous marriages are constructed, narrated, and understood? And finally, how does strategic multilingualism influence not only intra-household dynamics but also the very formation of these marriages? By addressing these questions, this study offers a new perspective on the cultural and communicative life of polygamous households in Uganda, one that situates language at the heart of family structure, identity, and adaptation.

## Literature review

### Polygamy in Uganda and in global perspective

Polygamy is a prevalent marital practice in East Africa, including Uganda, where it is deeply embedded in cultural traditions. In Uganda, approximately 6-11% of households are polygamous, while in Kenya, the range is 4-11% ( Johnson-Peretz et al., 2024). For instance, among the Acholi people in Uganda, polygamy has been a dominant pattern of sexual pairing, traditionally viewed as a beneficial institution rather than oppressive ( Amone, 2020). This practice is often justified culturally, with some communities viewing it as a means to accumulate wealth and social prestige ( Fenske, 2015).

Polygamy in Uganda and East Africa is intertwined with gender dynamics, often leading to increased risks for women. It has been shown that women in polygamous marriages are more likely to experience intimate partner violence compared to those in monogamous unions ( Ahinkorah, 2021). Additionally, polygamy is also associated with complex health dynamics, particularly concerning HIV transmission. In Uganda, polygamous families face unique challenges in HIV prevention and treatment, as the diagnosis of one member does not always lead to disclosure to others, complicating household dynamics ( Khanakwa et al., 2012; Johnson-Peretz et al., 2024).

The practice of polygamy is subject to ongoing debate, with traditionalists defending it as a cultural norm, while human rights activists criticize it as oppressive ( Amone, 2020; Fenske, 2015). In some regions, polygamy is seen as a means to ensure food security and child health, although these benefits are context-dependent and can vary significantly across different communities ( Lawson et al., 2015).

Studies from the Middle East and North Africa reveal striking parallels with the Ugandan case. A multi-country work shows that in Bedouin and Palestinian families, polygyny functions simultaneously as a kinship strategy and a marker of masculine status, often mediated through ritualised speech and honorifics ( Al-Krenawi and Slonim-Nevo, 2008). Likewise, Alhuzail and Lander (2021) document how adolescent Bedouin girls deploy metaphor and silent resistance to negotiate painful hierarchies within the compound household. These cross-regional findings bolster the argument that language is a core medium through which polygamous power is felt and contested.

Comparative economics points to three recurrent drivers of contemporary polygyny: wealth inequality, male-migration gaps, and legal pluralism ( Fenske, 2015). In Gulf states, oil wealth offsets bride-price inflation; in West Africa, rural land tenure fuels lineage expansion; and in war-affected regions such as northern Uganda and the Negev, demographic imbalance legitimises multi-wife unions ( Al-Krenawi, 2014). Embedding the Ugandan narratives inside this global matrix clarifies why selective multilingualism emerges: language capital becomes one more resource households mobilise to hedge against structural uncertainty, supporting the argument that linguistic exclusion is not a benign side effect but a psycho-social stressor requiring policy attention.

### Multilingualism in Uganda

The sociolinguistic landscape in Uganda is complex, with language use reflecting and influencing social structures and interactions. In Uganda, language can influence social cooperation and public goods provision, since it has been found that individuals contributed more in public goods games when using the national language, especially among those with strong local identities ( Clist and Verschoor, 2017). This suggests that language can activate cultural norms that affect social behavior and cooperation.

Multilingualism in Uganda is also intertwined with educational and social inequalities. While multilingualism is celebrated, it can sometimes obscure deeper socioeconomic disparities that it cannot address alone ( Duchêne, 2020). Many educational programs aim to incorporate multilingualism into literacy efforts, promoting local languages and cultural knowledge to enhance social equity ( Weidl et al., 2023). Additionally, the presence and type of multilingual written texts in Uganda reflect social stratification. Factors such as language policy and the status of speakers influence the availability and nature of these texts, which in turn affect the social standing of different languages and their speakers ( Reh, 2004). In Uganda, children in child-headed households utilize multilingual cultural resources, such as stories and proverbs, to navigate their social worlds ( Namazzi and Kendrick, 2014). This highlights the role of multilingualism in cultural transmission and social adaptation.

## Methodology

### Research design

This qualitative study employs narrative analysis to examine how language use reflects and constructs social hierarchies within polygamous marriages in Uganda. Such approaches are crucial in exploring how lived linguistic practices encode power, identity, and social roles, particularly in culturally specific marital arrangements like polygamy ( Mukama, 1998; Mugambi, 2014). The core of the research is a curated set of micro-narratives collected between 2023 and 2024 through SIDINL Newsletters, an online micro-storytelling platform. These narratives document lived experiences of individuals in polygamous and multilingual households, providing insight into how language functions as both a reflection of and a mechanism for negotiating power, identity, and emotional dynamics in such relationships.

The SIDINL (Specialized In-Depth Information & Newsletters) platform is a participatory storytelling tool that allows grassroots researchers in many countries, including Uganda, to collect and curate micro-narratives about pressing local issues. Between 2023 and 2024, a special edition of SIDINL focused on marriage and language, inviting contributions that documented everyday interactions within polygamous households across the country. Previous studies have emphasized the value of community-embedded qualitative research in capturing the complexity of family and gender roles in polygamous arrangements and have highlighted how such research can help uncover nuanced power dynamics often invisible in broader survey data ( Amone, 2020; Johnson-Peretz et al., 2024).

Local researchers gathered stories from husbands, co-wives, children, and in-laws in polygamous marriages. Narratives were collected in both oral (audio-recorded, later transcribed) and written forms, depending on literacy levels and regional practices. For this research, a total of 20 Ugandan languages are represented, covering Uganda’s major linguistic regions. This inclusive multilingual approach is supported by previous research highlighting the importance of language diversity in sociocultural studies, particularly in multilingual societies like Uganda where language choice often signals identity and social alignment ( Lorenz, 2019). These languages were selected based on regional diversity and the presence of documented polygamous households in the areas:

• Central Region: Luganda, Lugwere, Lusoga
• Western Region: Runyankore, Rukiga, Rutooro, Runyoro
• Eastern Region: Ateso, Lumasaba (Lugisu), Japadhola, Kumam
• Northern Region: Acholi, Lango, Alur, Madi
• West Nile Region: Lugbara, Aringa
• Karamoja Region: Karamojong
• Southwestern/Border Areas: Rufumbira, Rukonzo

This language selection allowed for a rich cross-linguistic and cross-cultural comparison of how polygamous family life is constructed and negotiated through language. Such comparative analysis aligns with findings that language practices vary significantly across Uganda’s ethnic groups and influence interpersonal dynamics and cultural values ( Tumwine and Ntozi, 2017). However, although the corpus spans 20 Ugandan languages, it under-represents highly mobile or peripheral groups (such as refugees, pastoral nomads, and women with restricted literacy in dominant tongues). Therefore, the present dataset does not yet capture narratives from Uganda’s displaced or nomadic populations, who frequently experience heightened linguistic precarity and social invisibility. Refugees settled along the West Nile frontier and pastoralist communities in Karamoja were beyond the geographic and budgetary reach of research, while many women with restricted literacy in lingua francas such as Luganda or English declined to contribute written stories, citing fear of misinterpretation. Similar sampling challenges have been reported in East-African studies of marginal language communities ( Mous, 2017) and in ethical discussions of site anonymity ( Nduna et al., 2022).

A total of approximately 200 narratives were collected, with about 10 stories from each of 20 different Ugandan languages. The narratives were contributed by local researchers embedded in various communities, and they represent a diverse range of participants including husbands, first and second wives, children (aged 12–25), and in some cases, extended family members. Participants were recruited through community organizations, local health centers, women’s groups, and word-of-mouth facilitated by SIDINL’s grassroots network. All participants gave informed consent, either orally or in writing, and pseudonyms were used to protect identities. Narratives were anonymized and reviewed by local curators to ensure cultural appropriateness and confidentiality, consistent with best ethical practices in qualitative research involving vulnerable populations and culturally sensitive topics, particularly around gender, marriage, and health ( Johnson-Peretz et al., 2024). Ethical oversight was provided by the ethics guidelines of the SIDINL initiative.

Names and identifying details were removed or pseudonymized by local researchers prior to analysis ( Montero-Sieburth, 2020). All participants gave informed consent, either written or oral, depending on literacy levels, for their stories to be recorded, shared, and potentially used for research and publication. For participants under the age of 18, informed consent was obtained from a parent or guardian, and verbal assent was sought from the minors themselves. Where written consent was not feasible due to literacy barriers, oral consent was documented by the local researchers.

### Data processing

The data processing phase involved both transcription and translation. Oral stories were transcribed in the original language and then carefully translated into English by bilingual researchers trained to preserve cultural idioms, metaphors, and nuance. Prior research has emphasized the challenges and importance of culturally sensitive translation in multilingual contexts, particularly in retaining the emotional tone and symbolic meaning of locally embedded expressions ( Lorenz, 2019). A hybrid coding framework was developed for analysis. Inductive coding was used to identify emergent themes such as jealousy, respect, favoritism, and emotional tone. Deductive coding applied a pre-established set of categories focusing on language use in conflict resolution, formality or informality of address, and the use of metaphor or proverb.

To ensure analytical rigor, inter-coder reliability checks were conducted among the research assistants. Three principal analytic approaches were used: discourse analysis, sociolinguistic analysis, and narrative analysis. Discourse analysis focused on how speakers used language to negotiate roles and power dynamics, including the use of pronouns, metaphors, and honorifics. This approach is supported by earlier studies which show that linguistic markers such as pronoun usage and metaphor in Ugandan languages are often employed to assert or challenge authority within family systems ( Mukama, 1998).

Even with this audit-trail workflow, some emotional resonance inevitably slips in cross-language transfer, since it can be a measurable drop in affective intensity when culturally gendered metaphors are flattened in translation ( Macht, 2018). Similar losses have been observed in Bedouin Arabic proverb research, where irony depends on vowel length and intonation that English cannot reproduce ( Nandram et al., 2025). Auto-ethnographic work on Korean diary narratives also shows that humour encoded in honorific play is almost impossible to replicate verbatim ( Yeom, 2024). Translation methodologists therefore urge researchers to preserve parallel texts, note decision points, and invite secondary re-reading to surface hidden meanings ( Zhao et al., 2025).

### Cross-linguistic comparisons

Sociolinguistic analysis examined the influence of dialect, code-switching, and language prestige on interpersonal interactions within polygamous households. These elements are known to reflect and mediate power asymmetries, especially where certain languages carry higher social capital, such as Luganda or English, often associated with education or urban status ( Nakayiza, 2016). Narrative analysis explored the structure and sequencing of the stories, looking at how individuals emphasized particular events, moral lessons, or relationship dynamics. This method has been shown to be particularly effective in capturing the voice and agency of marginalized participants within polygamous and gendered contexts ( Gumani and Sodi, 2009).

To contextualize findings, cross-linguistic comparisons were conducted to identify regional trends. For example, the analysis considered which language groups employed formal address more frequently when referring to co-wives or husbands, or how metaphor and indirect speech were used to either reinforce or resist social hierarchies. In some cases, switching from a local language to a more widely used lingua franca (such as Luganda or English) marked shifts in emotional tone or social intention. This phenomenon has been well documented in sociolinguistic studies, where such code-switching often signifies power shifts or emotional distancing during sensitive conversations ( Nakayiza, 2016).

While the methodology offers a unique, grounded view of the sociolinguistic dynamics of polygamous marriages in Uganda, it is not without limitations. The representation of languages and regions was uneven due to logistical and resource constraints. This issue has been noted in other regionally focused qualitative studies, where rural or marginalized groups are often underrepresented due to geographic or infrastructural barriers ( Tumwine and Ntozi, 2017). Some narratives may reflect self-censorship, especially regarding sensitive issues such as domestic violence or HIV status. This is consistent with findings from health and gender studies in Uganda, which highlight how stigma and household dynamics can limit disclosure, particularly in polygamous households ( Johnson-Peretz et al., 2024). Women and co-wives may downplay experiences of jealousy or abuse to preserve social harmony or due to fear of reprisal ( Gumani and Sodi, 2009). Moreover, despite efforts to retain cultural nuance, translation may have introduced interpretive bias or diminished the expressive depth of the original narratives. Nonetheless, this methodology enabled a culturally rooted, linguistically rich investigation of the everyday realities of polygamous marriage in Uganda, highlighting the central role of language in shaping and expressing power, intimacy, and identity within these complex domestic settings.

## Findings

### Language as a marker of hierarchy in polygamous marriages

Across the multilingual narratives collected ( Table 1), language consistently emerged as a powerful mechanism for expressing and reinforcing hierarchical roles within polygamous marriages. In particular, lexical choices, honorific forms, and referential strategies signaled the speaker’s position within the household and their relationship to others, especially between co-wives and between wives and husbands. This pattern was observable across most of the twenty language groups, though it took distinct forms shaped by regional cultural norms.

**Table 1.** Summary of language groupings and their dominant sociolinguistic features in polygamous storytelling.

Language region	Languages	Notable features in polygamous narratives
Central	Luganda, Lusoga, Lugwere	Use of polite honorifics, indirect speech for conflict, linguistic deference to senior wives
Western	Runyankore, Rukiga, Runyoro, Rutooro	High metaphor usage, emotional modulation through proverbs, formal respect language for in-laws
Eastern	Ateso, Lumasaba, Japadhola, Kumam	Switching between local language and English in marital conflict; strong idiomatic framing of jealousy
Northern	Acholi, Lango, Alur, Madi	Narrative focus on land, inheritance, and rivalry; use of euphemisms for emotional distance
West Nile/Karamoja	Lugbara, Aringa, Karamojong	Simple direct sentence structure; minimal switching; emphasis on fairness and justice among co-wives
Southwest Border	Rufumbira, Rukonzo	Use of Shona/Bantu metaphors; spiritual framing of marriage roles and family harmony

Source: Analysis from SIDINL Newsletters.

In Luganda and Lusoga in Uganda’s Central Region, wives frequently employed deferential address forms when referring to a senior wife (the first wife), especially in stories narrated by younger or later wives. One Lusoga speaker, for example, referred to her co-wife exclusively as the “mother of the elder children”, a respectful circumlocution that acknowledges the first wife’s primacy without using her name. This phrasing not only reflects linguistic politeness but also encodes social seniority. The narrator later explained, “It is not proper to write or say the first wife’s name when you are the second”, revealing an internalized linguistic etiquette that mirrors domestic hierarchy.

In the Runyankore-Rukiga narratives of southwestern Uganda, the distinction between wives was also emphasized through the use of particular kinship terms and metaphor. A second wife described her co-wife as “the one from the beginning,” a poetic term that frames the first wife as foundational and symbolically tied to origin and legitimacy. This language reflects broader Banyankore social norms, where age and order are key dimensions of status not only in marriage but in clan leadership and inheritance.

Interestingly, this pattern of linguistic deference was not only vertical (younger wife to senior wife) but also horizontal, in the ways husbands named or described their wives. In Ateso narratives from the Eastern Region, husbands sometimes used numerical ordering (“the first one”; “the second one”), while others emphasized emotional or functional roles: “the one who keeps peace in the home”. Such language subtly creates a hierarchy based on behavior, reinforcing internal competition or positioning wives as rivals for moral superiority, not just chronological order.

Moreover, in Acholi and Lango stories from the North, language use frequently revolved around land rights and childbearing, a key source of status among co-wives. In one Acholi narrative, a co-wife recalled being referred to by her husband as “the one who is strong at home”, which she understood to mean she had greater domestic influence despite being the second wife. The same speaker then contrasted this with her co-wife’s nickname “mother of the chief’s child”, noting that while she had household control, the first wife held status through her son’s position. These overlapping titles show how language both mirrors and negotiates layered status dimensions: power, lineage, and domestic management.

In multilingual households, especially in urban or mixed-ethnic settings, the choice of language itself carried status implications. In Kampala-based stories, for instance, some wives deliberately shifted to English or Luganda when addressing disputes in front of children or visitors, signaling education or urbanity. As one Lugwere-speaking wife noted, “When I use Luganda, he listens more, he thinks I am more serious. If I use our village tongue, he laughs.” This strategic language choice reflects a broader social valuation where “higher” languages (in terms of social capital) confer authority or rhetorical weight in household disputes.

These linguistic strategies, while diverse in form, point to a shared function: language is not merely a vehicle for communication but a tool for positioning, negotiating status, asserting legitimacy, and sustaining or contesting social order within the polygamous home. The narratives do not only describe lived experience; they perform it, using culturally coded speech to signal hierarchy and power. In many ways, the polygamous household becomes a linguistic microcosm of Uganda’s broader sociopolitical dynamics, where age, gender, and language operate as interlocking systems of stratification.

### Code-switching, multilingualism, and emotional dynamics

One of the most consistent and revealing patterns in the narratives was the strategic use of multiple languages within a single household to express or manage emotional dynamics, particularly in moments of conflict, affection, or negotiation. In multilingual Ugandan polygamous households, switching between languages was rarely arbitrary; it often carried social and emotional weight, marking shifts in power, intimacy, or tension between household members.

In households where the husband and co-wives came from different linguistic backgrounds, or where children were raised in urban multilingual environments, code-switching was often a way to navigate both private and public spaces. Many wives reported that their choice of language when addressing the husband depended on the emotional context. For instance, English or Luganda, a language widely associated with education and urban prestige, was commonly used when a wife wanted to assert authority, make a formal complaint, or demand fairness, particularly in front of children or extended family members. In contrast, local mother tongues were often used in intimate or conciliatory moments, such as resolving a dispute quietly or making emotional appeals.

This deliberate shifting between languages often signaled emotional boundaries or levels of seriousness. Several women reported that their husbands paid more attention or “took things more seriously” when issues were raised in a language perceived as more formal or public. For example, one woman from a Lugbara-speaking household explained that when she felt disrespected, she would switch from their usual local language to English to “change the tone” and “put him in the right frame of mind.” This switch was not only functional, it was performative, signaling a change in status or the emotional gravity of the conversation.

Intra-wife relationships also revealed significant code-switching behavior. Co-wives would often shift between politeness and passive aggression depending on language choice. In some cases, wives who shared a common mother tongue would use a different lingua franca, such as Luganda or Kiswahili, when speaking in front of others, especially during subtle disagreements. This shift often served to mask tension from children or elders, or to perform civility while still conveying veiled criticisms.

Children in these households also displayed strategic multilingualism, especially when navigating competing loyalties. In several narratives, young people described using the mother tongue of the wife they were closest to when seeking favors or comfort but switching to a “neutral” or dominant household language (often English or Luganda) when addressing both parents together. This choice was more than convenience, it reflected a negotiation of family identity, alliance, and emotional safety.

What emerged clearly from the analysis is that multilingualism within polygamous households is not merely a byproduct of Uganda’s linguistic diversity, it is an emotional and relational resource. Language choice and code-switching serve as subtle tools for managing vulnerability, asserting identity, and mediating conflict. In this way, language becomes a soft form of power, one that shapes not only what is said, but how it is felt and received.

These private household narratives reveal a deeper layer of language use: one tied intimately to emotional life. In polygamous families, where relational hierarchies are sensitive and constantly negotiated, multilingualism offers not just a means of expression but a flexible repertoire for navigating complexity, linguistically, socially, and psychologically.

### Regional variation and cultural framing

While linguistic hierarchies and emotional code-switching were observed across Uganda, the way polygamous marriage was framed in storytelling varied notably by region and language group. These variations reveal how cultural values, such as seniority, fertility, peacekeeping, or wealth, shape how polygamy is understood and narrated within specific communities.

In the Western Region, particularly among Runyankore and Rutooro speakers, narratives often framed polygamy through metaphor and proverb, emphasizing restraint, dignity, and the preservation of harmony. Wives who shared their experiences frequently used traditional sayings or moral framing to describe household dynamics. Even when tension or rivalry existed between co-wives, the language often highlighted endurance or wisdom, portraying the first wife as a stabilizing matriarch and later wives as needing to “learn patience” and “respect the home they found.” This reflects broader Western Ugandan norms that associate virtue with self-control and social balance, especially among women.

In contrast, Northern Uganda, including Acholi and Lango narratives, emphasized land, children, and inheritance as central themes in the framing of polygamous marriage. Stories here were more likely to discuss legal and customary issues, such as whose children inherit what land, or how a man’s estate is divided among his wives. Co-wives’ identities were closely tied to the number and gender of their children, especially sons. This framing created a narrative lens where success and power in the marriage were measured less by emotional dynamics and more by tangible assets and social continuity. One common narrative pattern involved the wives subtly competing through their children’s achievements, or the amount of land “claimed” through them, reinforcing a patrilineal logic that structured household power.

In the Eastern Region, particularly among Ateso and Lumasaba speakers, narratives often carried a more open and dialogical tone. Here, conflict was frequently narrated with directness, sometimes even humor, and less concern with masking rivalry through metaphor. Several wives openly discussed jealousy, unfairness, or favoritism in a tone that blended critique with self-awareness. One woman described “sharing love like a market tomato”, a striking metaphor used to express the fragmentation of affection across wives. This culturally grounded storytelling reflected a more pragmatic view of polygamy, less about symbolic roles and more about emotional negotiation and daily fairness.

Among West Nile language groups, including Lugbara and Aringa, the framing of polygamous marriage emphasized justice and fairness. Many narratives focused on the husband’s ability to “balance” time, resources, and emotional attention. Wives evaluated their own status based on whether they felt treated equitably rather than on their order of marriage. This aligns with regional moral values that emphasize distributive justice and the public perception of fairness. Unlike in other regions, the narratives here made fewer references to spiritual roles or metaphysical hierarchy, focusing instead on material equity and social standing within the village.

Lastly, among border communities like the Rukonzo and Rufumbira speakers, the framing of marriage often intertwined with spirituality and religious metaphors. Stories included references to blessings, curses, and divine tests. Several wives interpreted their position in the household through a spiritual lens, with marriage challenges viewed as trials of faith or fate. This metaphysical dimension added a unique layer to the understanding of hierarchy and resilience within polygamous unions, where success was not only social but spiritual.

These regional differences do not merely reflect variation in language use, they represent culturally embedded worldviews about what it means to share a husband, raise children communally, and navigate competition in intimate space. In some communities, polygamy was framed as a moral trial; in others, as a financial or territorial negotiation; and in still others, as a personal journey requiring strategic communication. The way stories were told, whether poetic or pragmatic, symbolic or straightforward, illuminates how deeply intertwined language, culture, and marriage structures are across Uganda’s diverse linguistic landscape.

Together, these patterns reveal that polygamy in Uganda is not a singular institution but a set of regionally and culturally specific practices, each mediated and expressed through distinctive linguistic choices. Understanding these framings is essential not only for interpreting individual narratives but also for appreciating how language carries cultural ideology, shaping how people perceive their roles and rights within complex family systems.

### Selective multilingualism and language-based polygamous strategies

Beyond household dynamics and emotional communication, several narratives revealed a more strategic function of multilingualism in shaping the very formation of polygamous marriages. In a number of cases across Uganda, the choice of a second or third wife was influenced, explicitly or implicitly, by the language she spoke. This practice, which can be termed selective multilingualism, reflects how language is valued not only as a communicative tool but also as a form of social capital that extends beyond the household.

In urban centers such as Kampala, Mbale, and Fort Portal, men frequently described choosing wives who spoke a dominant regional or national language, such as Luganda, English, or Swahili, not just for ease of communication, but for integration into broader social and economic networks. For example, one husband from a Langi-speaking background explained that his decision to marry a Luganda-speaking woman was partly driven by his recent migration to the Central Region. “She could speak for me,” he said, referring to his wife’s ability to negotiate with landlords, neighbors, and school officials in Luganda. Here, the wife’s language offered access to local services and helped the family gain legitimacy in an unfamiliar sociolinguistic environment.

In borderland regions, where cross-border trade and movement are common, language choice also played a critical role. Among Rufumbira speakers in southwestern Uganda, narratives indicated that men strategically married women who were bilingual in both Ugandan and Rwandan dialects, facilitating smoother cross-border business. In this context, a wife’s language abilities were seen not just as a household asset, but as a gateway to mobility and trade. Similarly, in the West Nile Region, men engaging in small-scale commerce in South Sudan or the DRC preferred wives who spoke Lugbara or Aringa fluently, as these languages offered cultural legitimacy in both directions of the border.

In rural-to-urban migration contexts, language often served as a bridge or barrier to adaptation. Several urban-based men who originally came from monolingual rural communities described choosing an “urban wife” who could navigate bureaucratic systems and institutions. These women, often fluent in English or Luganda, became key facilitators of upward mobility. As one narrative put it, “My first wife knew our land, but my second wife knew the city.” The linguistic contrast here reflected more than geography, it mapped onto differing forms of expertise, with one wife rooted in customary land and social life, and the other operating as a cultural intermediary in urban space.

Interestingly, this practice of language-based marriage choice was not always symmetrical. While men strategically valued a wife’s multilingualism, women rarely cited the husband’s language as a primary reason for accepting marriage proposals. Instead, women spoke more about emotional compatibility, economic security, or kinship alliances. However, some women did express a sense of linguistic vulnerability, particularly when marrying into households that spoke unfamiliar languages. These women reported feelings of isolation or dependence, especially when co-wives or in-laws used a shared language the newcomer could not understand. In such cases, lack of multilingualism could deepen feelings of exclusion or powerlessness, subtly reinforcing intra-household hierarchies.

What these narratives reveal is that multilingualism in Uganda operates not only within households but also before and beyond them, shaping how polygamous marriages are formed, how couples adapt to changing geographies, and how language becomes tied to access: access to land, to urban systems, to education, to legitimacy. The linguistic profile of a potential wife, then, is not merely personal, it becomes instrumental, part of the long-term survival and expansion strategy of the family unit.

This form of selective multilingualism complicates simple assumptions about love, tradition, or desire in marriage choices. It introduces a socioeconomic and geopolitical logic where language becomes a resource, valued not for its beauty or heritage alone, but for what it enables in practical terms. As Uganda continues to urbanize and regional migration intensifies, such patterns may become even more pronounced, further entrenching language as both a symbolic and functional currency in the evolution of polygamous households.

## Discussion

The analysis of these 200 multilingual narratives collected through the SIDINL platform between 2023 and 2024 revealed how language intricately shapes the structure and experience of polygamous marriages in Uganda. Language functioned not merely as a medium of communication, but as a powerful social instrument for expressing hierarchy, managing emotional relationships, and even influencing partner selection. Across all 20 languages studied, individuals used linguistic choices, such as honorifics, metaphors, and strategic code-switching. to negotiate status, assert identity, and maintain or contest household balance.

The findings also demonstrated that language operates both within and beyond the intimate sphere. Selective multilingualism emerged as a pattern in which certain languages, due to their associations with urban life, commerce, or political access, shaped the very formation of polygamous unions. Regionally, narratives showed divergent cultural framings of polygamy: from moral endurance in the West to inheritance-based competition in the North, and spiritual interpretations in the borderlands. These patterns point to a deeply embedded relationship between linguistic practice and the broader sociocultural, economic, and spiritual dimensions of family life in Uganda.

### Language as a social instrument in family systems

These findings reinforce longstanding sociolinguistic arguments that language is not merely a passive reflection of social structure, but an active force in shaping it ( Gordon, 2011; Mukama, 1998). In the polygamous Ugandan households represented here, language structured interpersonal hierarchies, particularly between co-wives and between wives and husbands. Honorific forms and euphemistic naming practices, such as calling a first wife “mother of the elder children” or “the one from the beginning”, not only marked respect but signaled social rank and emotional positioning. These discursive strategies are not arbitrary; they are embedded in cultural expectations around age, legitimacy, and marital order, particularly in communities like the Banyankore and Basoga where lineage and seniority carry significant weight ( Amone, 2020).

The use of metaphor and circumlocution, especially in Runyankore, Rutooro, and Luganda narratives, further illustrates how language serves to maintain social cohesion while indirectly negotiating tension. As Reh (2004) and Lorenz (2019) have shown, metaphor in Ugandan oral discourse often functions as both a face-saving strategy and a coded means of critique. This was evident in how wives spoke about rivalry or dissatisfaction, using poetic language to maintain politeness while asserting personal insight or grievance.

Moreover, language was used to reinforce emotional power dynamics. Wives described using English or Luganda when asserting authority in disputes, while switching to their mother tongue when seeking empathy or reconciliation. This aligns with Nakayiza’s (2016) findings that language prestige in Uganda is closely tied to perceptions of modernity, seriousness, and power. Thus, even within a single marriage, the strategic choice of language could recalibrate emotional tone, shift power temporarily, or reposition the speaker socially.

In this way, language served as a performative act, not just describing reality, but actively constituting it ( Austin, 1962; Oleksy, 2019). When a wife chooses a more formal language to demand respect, or when a husband assigns nicknames that encode power and affection, they are not only reflecting household dynamics, but they are also shaping them in real time. This performativity is particularly salient in polygamous households where roles are complex and constantly renegotiated.

Ultimately, this research affirms that linguistic practice in family life is deeply ideological. It enacts gendered, generational, and cultural expectations, often without needing to be explicit. The nuanced ways Ugandans use language in polygamous contexts show how speech is never neutral, it is always embedded in structures of power, obligation, and identity.

### Multilingualism, mobility, and strategic marriage choices

The emergence of selective multilingualism in the findings points to a significant sociocultural shift in how language is valued within marriage, particularly in Uganda’s increasingly mobile and urbanizing society. Rather than language functioning solely within the household to manage relationships, it also plays a role in shaping the household, especially in how polygamous unions are formed, maintained, or strategically expanded. In many of the SIDINL narratives, men described choosing wives not just based on affection or fertility, but on the perceived value of a woman’s linguistic repertoire in accessing new social, economic, or geographic spaces. This supports scholarship showing that language choice reflects broader strategies of group survival and identity negotiation under changing social and economic conditions ( Mous, 2017). This also reflects what Bourdieu (1991) termed the “symbolic capital” of language, where certain tongues carry the ability to unlock institutional legitimacy, employment, and upward mobility.

This logic was particularly evident in stories from migrant or peri-urban families. For instance, Luganda and English were commonly cited as desirable languages for a second or third wife, especially in Kampala or central towns, due to their association with education, administrative fluency, and social prestige. This matches prior findings that in Uganda’s multilingual cities, language is often a proxy for class and education, where speaking a dominant or elite language grants perceived competence and access to urban resources ( Nakayiza, 2016; Clist and Verschoor, 2017). In one narrative, a husband framed his Luganda-speaking wife as a “public interface,” able to liaise with landlords and schools. Her linguistic skills were framed not as secondary traits but as instrumental contributions to the household’s survival and social legitimacy in the city.

At the same time, rural and border narratives introduced a different linguistic logic, where multilingualism was valued for geopolitical access, such as ease of cross-border trade or integration into new ethnic or linguistic networks. Among Rufumbira- and Lugbara-speaking families, wives who could navigate Rwandan or Congolese dialects were seen as vital to expanding family commerce or smoothing social relations across boundaries. This reflects an under-researched aspect of polygamous strategy: how marriage becomes a tool of regional integration, and how women’s linguistic fluency becomes tied to broader household survival and prosperity ( Gumani and Sodi, 2009).

These patterns challenge romantic or static views of polygamy as purely customary. Instead, they suggest that polygamous marriage is evolving into a pragmatic system of adaptive expansion, a flexible architecture for accessing different cultural and economic capitals ( Ikamari and Agwanda, 2020). Multilingualism, in this context, becomes not only an internal household asset, but an external strategy for navigating uncertainty in a rapidly changing Uganda. This aligns with recent anthropological critiques that African marriage systems are becoming increasingly “instrumentalized” in response to labor migration, urbanization, and shifting gender economies ( Lawson et al., 2015).

However, this relationship also reproduces new forms of inequality. While men exercised choice based on the strategic value of language, women often entered such unions without the same agency. Narratives from wives who felt isolated due to language barriers reflect how lack of access to the household’s dominant language can lead to emotional marginalization and reinforce asymmetries between co-wives. This tension underscores Duchêne’s (2020) caution that while multilingualism can offer mobility, it can also mask deepening inequalities, especially when language becomes a gatekeeping tool within intimate relationships.

In sum, language in polygamous marriage is not merely a communicative medium but an evolving currency of mobility and survival. It reflects not only personal identity but the household’s broader strategies for adaptation, legitimacy, and access, whether to land, institutions, or the public sphere. Multilingualism in this sense is not just social, it is economic, spatial, and political.

### Cultural diversity and the limits of policy approaches

The diversity of language practices observed here underscores a critical challenge for policymakers and social institutions in Uganda: standardized approaches to language, gender equity, and family structures often fail to reflect the lived realities of multilingual and culturally embedded polygamous households. The data gathered through SIDINL highlights how deeply localized and flexible social systems can be, especially when shaped by linguistic plurality. While Uganda’s education and language policies have increasingly emphasized multilingual literacy and local language inclusion ( Weidl et al., 2023), these initiatives often operate within formal institutional spaces and do not adequately account for how language functions in everyday domestic and emotional life.

The findings reveal, for instance, that language hierarchies are often reproduced not through state policy, but through intimate micro-practices: in how a wife is named, which language is used during conflict, or how children align themselves linguistically in blended households. These dynamics fall outside the scope of most formal gender and language equality policies, yet they significantly shape social status and emotional wellbeing. As Reh (2004) and Namazzi and Kendrick (2014) have shown, written multilingualism in Uganda may appear inclusive on the surface, but the symbolic power of dominant languages continues to marginalize those whose linguistic capital is not institutionally recognized. This is mirrored in the domestic sphere, where prestige languages like English and Luganda can become tools of both empowerment and exclusion.

Moreover, many gender equality frameworks promoted by NGOs and state bodies focus on combating the structural risks of polygamy, such as intimate partner violence or inheritance inequality ( Ahinkorah, 2021; Johnson-Peretz et al., 2024), without accounting for how language mediates those risks in culturally specific ways. For example, the indirect language used by some wives to express dissatisfaction may be misread as consent or harmony by external actors unfamiliar with the cultural weight of euphemism or silence in Ugandan storytelling traditions ( Mukama, 1998). Likewise, emotional distress or marginalization rooted in linguistic isolation may go unaddressed in interventions that assume communication breakdowns are primarily economic or behavioral.

Promoting equity in multicultural polygamous households calls for a specific agenda that can be both low-cost and evidence-based: first, establish co-wife mediation circles led by bilingual para-legal facilitators so that every wife can articulate grievances in her strongest language ( Zucker, 2021); second, create district-level community-interpreter rosters, funded through modest stipends, to accompany women during land-title negotiations, maintenance hearings, or protection-order applications ( Zucker, 2021); third, adopt school language-inclusion charters that legitimise children’s heritage tongues and undercut status hierarchies reproduced by monolingual classrooms; and finally, incorporate explicit language-rights clauses into ongoing family-law reforms, so that legal protections address gender and linguistic marginalisation in tandem ( Oyugi, 2017).

These limitations point to a broader issue in policy design: the need for grounded, ethnographically informed approaches that take local linguistic practices seriously, not just as communication methods, but as social actions loaded with power, history, and meaning. A co-wife’s silence in a multilingual household may be an act of survival, deference, or protest, depending on the language context. Treating language merely as a neutral tool risks obscuring the very dynamics that drive household inequality or cohesion ( Mazrui and Mazrui, 1993; Duchêne, 2020).

Furthermore, as Uganda continues to urbanize and its sociolinguistic landscape becomes more complex, the state’s language policies will face increasing pressure to adapt. Already, strategic multilingualism is reshaping marriage patterns, family mobility, and even economic access, as this study demonstrates. Language planning cannot remain limited to schools and media if it hopes to address inequality at its roots. Instead, it must expand to include informal and domestic spheres, where language plays a profound role in shaping lived experiences ( Namazzi and Kendrick, 2014).

Viewing language as an emotional-social force requires public services to treat mother tongues as assets, not obstacles, when designing inclusive programmes ( Trudell¸2016). Multilingual counselling rooms that permit free code-switching have doubled client-retention rates in pilot mental-health clinics ( Lee, 2017). Taken together, these evidence-based interventions signal that language-responsive services cannot remain isolated pilot schemes, but they must feed into a broader rethinking of Uganda’s celebrated yet complex multilingual landscape, one that attends not only to which languages are spoken, but to how language is lived, negotiated, and emotionally felt in everyday life. Therefore, while Uganda’s multilingualism is often celebrated, its complexity demands more than symbolic inclusion or top-down frameworks. It requires attentiveness to how language is lived, negotiated, and felt, especially in polygamous households where identities, power, and belonging are continuously shaped by what is said, and by the language in which it is said ( Ikamari and Agwanda, 2020).

### Directions for future research

This study makes a significant contribution to the understanding of how language intersects with social structure, gender, and cultural practice in Uganda’s polygamous households. By analyzing multilingual narratives, it highlights how language operates not only as a tool for communication but as a system of social positioning, emotional expression, and strategic adaptation within marriage ( Mous, 2017; Mazrui and Mazrui, 1993). The use of grassroots micro-narratives from the SIDINL platform further demonstrates the value of locally generated qualitative data in capturing nuanced household dynamics often overlooked by surveys or institutional studies.

One of the key strengths of this research lies in its comparative, cross-regional scope. The study offers insights into how different cultural groups in Uganda frame polygamy. through metaphor, morality, land-based rivalry, or spiritual symbolism, and how these framings are embedded in language. It also surfaces the emerging phenomenon of selective multilingualism, where language plays a role in marriage formation and mobility, suggesting a shift toward more strategic, future-oriented family planning ( Ikamari and Agwanda, 2020).

Nevertheless, the study has limitations. Regional representation was uneven, with fewer narratives from highly mobile or marginal communities such as refugees or nomadic groups. Some degree of self-censorship is likely, particularly around sensitive issues such as marital conflict or emotional exclusion. Additionally, while translation was handled with care, some cultural depth may have been lost in moving from local languages to English ( Lorenz, 2019).

Future research could explore how children in multilingual polygamous households negotiate identity, belonging, and language loyalty over time ( Namazzi and Kendrick, 2014). Longitudinal studies may also reveal how multilingual family structures adapt as Uganda continues to urbanize and integrate regionally. More focused investigation into how language influences access to education, healthcare, and legal systems within polygamous families would further deepen our understanding of language as a determinant of social equity ( Omoeva and Hatch, 2022).

## Conclusion

This study has shown that language plays a central, often underestimated role in shaping the social fabric of polygamous marriages in Uganda. Through the analysis of multilingual narratives from across the country, it became clear that linguistic choices, ranging from honorifics to code-switching, actively reflect, reinforce, and sometimes challenge power dynamics, emotional bonds, and household hierarchies. Language is not simply a medium of expression; it is a social strategy that enables negotiation of identity, status, and belonging in complex marital settings. The diversity of regional storytelling further reveals how polygamy is not a monolithic institution, but one deeply adapted to cultural, linguistic, and geographic contexts.

As Uganda continues to navigate rapid social and linguistic change, understanding how language operates within families, especially non-normative or plural family structures, is essential. This research calls for greater attention to domestic language practices in both academic inquiry and policy design. Whether shaping household roles, influencing partner selection, or granting access to social systems, language remains a powerful force in the lived experience of marriage. Recognizing and valuing this complexity is crucial for creating more inclusive frameworks that reflect the realities of multilingual, multicultural societies.

### Ethical considerations

This study is based on secondary data drawn from the SIDINL platform, where narratives were collected between 2023 and 2024 as part of a participatory research initiative on language and marriage in Uganda. The author did not directly interact with participants or collect any new data.

Ethical approval for the current study is not required, as it involves the secondary use of fully anonymized qualitative data that was originally gathered under the SIDINL initiative. The original data collection followed internal ethical guidelines developed by SIDINL, including informed consent procedures, and pseudonymization of names. Because individual identifiers were removed before the dataset was accessed by the author, and no re-contact with participants occurred, the study complies with guidelines for secondary use of non-identifiable human data ( Tripathy, 2013; Lopez and Vann, 2021; Nduna et al., 2022).

In line with the Declaration of Helsinki, the study poses minimal risk to participants and maintains full respect for privacy and dignity.

All participants gave informed consent, either written or oral, depending on literacy levels, for their stories to be recorded, shared, and potentially used for research and publication. For participants under the age of 18, informed consent was obtained from a parent or guardian, and verbal assent was sought from the minors themselves. Where written consent was not feasible due to literacy barriers, oral consent was documented by the local researchers.

The narratives used in this study do not involve sensitive personal data or topics likely to cause harm, and the risk of re-identification is minimal. This research follows best practices for working with vulnerable populations, particularly in relation to gender, language, and cultural context ( Nowrouzi-Kia et al., 2020; Sony, 2025). Ethical oversight for data collection was guided by the SIDINL initiative’s internal protocols, which include community review and culturally appropriate anonymization strategies ( Montero-Sieburth, 2020). No additional data were collected directly by the authors beyond those shared through the SIDINL platform.

## Data Availability

The data utilized in this research consist of private, secondary narratives collected through the SIDINL platform between 2023 and 2024. These narratives are not publicly available due to their sensitive cultural content and the ethical obligation to maintain participant confidentiality ( Palmer, 2008). All data were collected with informed consent under conditions that specified restricted access and anonymization for scholarly use only ( Lopez and Vann, 2021). In line with ethical guidelines and best practices for research involving potentially vulnerable populations, full access to the raw narratives is limited ( Nowrouzi-Kia et al., 2020). However, researchers who wish to request access to the data for academic or verification purposes may do so through the SIDINL team at www.sidinl.com or to the corresponding author ( ekyomugisha@proton.me). Requests will be reviewed on a case-by-case basis to ensure that data use aligns with participant consent agreements, community ethics protocols, and data protection standards. While the raw data cannot be made openly accessible, metadata and analytical frameworks developed from the narratives (e.g., coding categories, thematic summaries, language grouping structures) are available upon reasonable request from the corresponding author. This approach seeks to balance transparency and reproducibility with ethical responsibility and cultural sensitivity ( González-Arias et al., 2024).
