# Supplementary material for: Voices of Plurality: Linguistic Diversity and Social Interactions in Ugandan Polygamous Marriages
Source: F1000Res. 2025 Aug 4;14:453. Originally published 2025 Apr 22. [Version 2] doi: 10.12688/f1000research.163648.2 (PMC12449685; doi:10.12688/f1000research.163648.2)
Supplement: Peer review history

# Reviewer report: Reviewer response for version 2

**DOI:** 10.5256/f1000research.185980.r413278

**Published:** 2025-09-19

**Authors:** David Oluwabukunmi Ogunbiyi

**Affiliations:** 1 Redeemer's University, Ede, Osun, Nigeria

This study investigates the connection between Ugandan family dynamics, sociolinguistics, and polygamous marriages. It investigates how language shapes intimacy, power, and identity in polygamous families using sociolinguistic theory, narrative analysis, and cross-regional comparisons. The study's 200 micro-narratives in 20 languages provide the results depth and variety. It introduces chosen multilingualism as a factor in marriage formation and draws attention to the relationship between language use and polygamy. The study offers substantial data and a cultural foundation while connecting findings to studies from the Middle East and West Africa. It provides practical suggestions for practice and policy, including multilingual counselling rooms, community interpreter rosters, and bilingual paralegal facilitators. There is need for improvement, nonetheless, in a few areas, including gendered agency, quantitative balance, sample gaps, terminology explanations, and better formatting.

Is the work clearly and accurately presented and does it cite the current literature?

Yes

If applicable, is the statistical analysis and its interpretation appropriate?

Yes

Are all the source data underlying the results available to ensure full reproducibility?

Yes

Is the study design appropriate and is the work technically sound?

Yes

Are the conclusions drawn adequately supported by the results?

Yes

Are sufficient details of methods and analysis provided to allow replication by others?

Yes

Reviewer Expertise:

My area of research centred on socio-religious, issues, religion, contemporary studies etc

I confirm that I have read this submission and believe that I have an appropriate level of expertise to confirm that it is of an acceptable scientific standard, however I have significant reservations, as outlined above.

# Reviewer report: Reviewer response for version 1

**DOI:** 10.5256/f1000research.180036.r380442

**Published:** 2025-06-25

**Authors:** Nuzha Allassad Alhuzail

**Affiliations:** 1 Sapir Academic College, Ashkelon, Israel

**Review of the Manuscript: "Voices of Plurality: Linguistic Diversity and Social Interactions in Ugandan Polygamous Marriages"**

 I would like to begin by thanking the editors for the opportunity to review this manuscript, which offers a highly original and insightful contribution to the existing literature. The study presents a novel perspective on familial dynamics in multilingual societies through the lens of polygamous households in Uganda. Its methodological richness and analytical depth make it a valuable addition to interdisciplinary discourse on language, gender, and family life.

**Strengths and Scholarly Contribution**

 This article provides an innovative and compelling contribution to our understanding of family relations in multilingual societies by exploring the sociolinguistic dimensions of polygamy in Uganda. Drawing on a rich dataset of 200 micro-narratives across 20 local languages, the study enables a deep cross-cultural and cross-regional analysis. The manuscript effectively demonstrates that language is not merely a vehicle of communication but serves as a powerful social, emotional, and strategic resource. Notably, it highlights the use of honorifics, metaphor, and code-switching to express status, manage tension, articulate intimacy, and negotiate household hierarchies.

 Moreover, the study introduces and theorizes the phenomenon of “selective multilingualism,” showing how linguistic fluency influences partner selection and access to urban and economic spaces. These findings are strongly grounded in contemporary theoretical frameworks and reflect a well-executed qualitative methodology. The integration of linguistic, gendered, cultural, and geopolitical analyses renders this study both intellectually rich and practically relevant.

**Recommendations for Improvement**

 Despite its significant strengths, the manuscript would benefit from several clarifications and expansions. First, there is a slight imbalance in the regional and communal representation of the narratives, with underrepresentation of highly mobile or peripheral populations. It is recommended that future work include voices from refugee communities, nomadic groups, and women with limited access to dominant languages.

 Second, while the manuscript acknowledges the challenges of translation, a more robust discussion of the potential loss of emotional and cultural nuance, particularly in metaphor and irony, would enhance the transparency and reflexivity of the study. Additionally, although the role of language as a form of power is well illustrated, the policy and educational implications of these findings could be developed further. For example, how might the understanding of language as an emotional-social force inform culturally responsive public policy?

 Lastly, a discussion of practical strategies for promoting both gender and linguistic justice in the context of polygamous marriage within multicultural settings would be a valuable extension of the analysis.

 The literature review is relatively limited in scope and could be meaningfully expanded. In particular, it would benefit from a broader discussion of polygamy as a global phenomenon, including its structural drivers and socio-psychological consequences. I recommend incorporating relevant scholarship by Allassad Alhuzail and Al-Krenawi, whose work addresses the intersections of polygamy, marginalization, and cultural context, and could provide a valuable comparative lens for this study.

 ( refer to 1, 2 and 3 )

Is the work clearly and accurately presented and does it cite the current literature?

Partly

If applicable, is the statistical analysis and its interpretation appropriate?

Not applicable

Are all the source data underlying the results available to ensure full reproducibility?

No source data required

Is the study design appropriate and is the work technically sound?

Yes

Are the conclusions drawn adequately supported by the results?

Yes

Are sufficient details of methods and analysis provided to allow replication by others?

Partly

Reviewer Expertise:

My research focuses on traditional societies, with a particular emphasis on the lived experiences of women in polygamous family structures. As a qualitative researcher, I employ narrative and ethnographic methods to explore how gender, kinship, and power are constructed and negotiated within culturally embedded frameworks. I am especially interested in how social norms, religious values, and indigenous knowledge systems shape women's roles, agency, and resistance in patriarchal contexts. My work contributes to the broader fields of gender studies, family sociology, and qualitative social research by offering context-sensitive insights into the intersection of tradition, inequality, and social change.

I confirm that I have read this submission and believe that I have an appropriate level of expertise to confirm that it is of an acceptable scientific standard, however I have significant reservations, as outlined above.

## References

[1] : “I Wish he Were Dead.” The Experience of Loss among Young Arab-Bedouin Women in Polygamous Families. *Affilia* .2023;38(1) : 10.1177/08861099221075899 75-90 10.1177/08861099221075899

[2] : The Experience of Bedouin-Arab Adolescent Girls in Polygamous Families. *Journal of Feminist Family Therapy* .2021;33(2) : 10.1080/08952833.2020.1755164 131-156 10.1080/08952833.2020.1755164

[3] : Being a Girl in a Polygamous Family Implications and Challenges. *Child and Adolescent Social Work Journal* .2020;37(1) : 10.1007/s10560-019-00623-w 97-107 10.1007/s10560-019-00623-w

# Response

**Published:** 2025-07-31

**Authors:** Elizabeth Kyomugisha

**Affiliations:** Kampala International University, Kampala, Central Region, Uganda

**Reviewer Comment #1**

**“Despite its significant strengths, the manuscript would benefit from several clarifications and expansions. First, there is a slight imbalance in the regional and communal representation of the narratives, with underrepresentation of highly mobile or peripheral populations. It is recommended that future work include voices from refugee communities, nomadic groups, and women with limited access to dominant languages.”**

 Response: Thank you for highlighting the under-representation of highly mobile and peripheral populations in this dataset. To make this limitation explicit, I have (i) added clarifying sentences about sampling limitations. This explains why refugee, nomadic, and low-literacy women’s narratives were not captured. I have also referenced Mous (2017) and Nduna et al. (2022) to situate this limitation within current discussions of linguistic marginality and ethical data practices. I hope these additions satisfactorily address the reviewer’s concern.

**Reviewer Comment #2**

**“Second, while the manuscript acknowledges the challenges of translation, a more robust discussion of the potential loss of emotional and cultural nuance, particularly in metaphor and irony, would enhance the transparency and reflexivity of the study. Additionally, although the role of language as a form of power is well illustrated, the policy and educational implications of these findings could be developed further. For example, how might the understanding of language as an emotional-social force inform culturally responsive public policy?”**

 Response: I agree that further reflexivity on translation loss and stronger policy links would enrich the manuscript. I have therefore inserted a new paragraph in data processing that synthesises recent scholarship on metaphor and irony fidelity and explains the parallel-text audit trail. I also expanded the discussion on “Policy and Educational Implications” to outline culturally responsive interventions, clarifying how recognising language’s affective power can inform both public policy and educational practice. I added pertinent references for this purpose, and I trust these revisions address your concerns regarding transparency, reflexivity, and applied relevance.

**Reviewer Comment #3**

**“Lastly, a discussion of practical strategies for promoting both gender and linguistic justice in the context of polygamous marriage within multicultural settings would be a valuable extension of the analysis.”**

 Response: I appreciate the suggestion to translate this analysis into actionable guidance. Accordingly, I have added a dedicated paragraph under the discussion that outlines concrete, evidence-based measures (co-wife mediation circles, district community-interpreter rosters, school language-inclusion charters, and language-rights clauses in forthcoming family-law reforms). This new text demonstrates how recognising language as an emotional-social force can drive gender-equitable and linguistically safe practices in multicultural polygamous households.

**Reviewer Comment #4**

**“The literature review is relatively limited in scope and could be meaningfully expanded. In particular, it would benefit from a broader discussion of polygamy as a global phenomenon, including its structural drivers and socio-psychological consequences. I recommend incorporating relevant scholarship by Allassad Alhuzail and Al-Krenawi, whose work addresses the intersections of polygamy, marginalization, and cultural context, and could provide a valuable comparative lens for this study.”**

 Response: Thank you for urging a broader, cross-regional framing. I have added the perspective of “Polygamy in Global Perspective” in literature review that situates Ugandan patterns alongside Middle-Eastern, West-African, and Gulf findings; (ii) integrated some new paragraphs that discuss structural drivers and psycho-social consequences; and (iii) added three new references. These insertions explicitly connect this concept of selective multilingualism to global scholarship on patri-kinship strategy and emotional wellbeing. I trust the expanded review now meets the depth and comparative breadth you recommended.

# Reviewer report: Reviewer response for version 1

**DOI:** 10.5256/f1000research.180036.r380440

**Published:** 2025-05-09

**Authors:** Omar Hazaymeh, Sufian Hazaymeh

**Affiliations:** 1 Al-Balqa Applied University / Al-Huson University College, Irbid, Jordan; 2 Independent Scholar, na, Jordan

This paper examines how linguistic diversity shapes social interactions within polygamous marriages in Uganda. Using ethnographic methods, the study highlights how co-wives, husbands, and children navigate multilingual environments to express identity, negotiate relationships, and manage household dynamics. It shows that language use within these families is not merely functional but carries deep social meanings related to power, solidarity, and emotional connection. The research reveals that multilingualism can both unite and divide members of the same household, depending on shifting alliances and personal relationships. Overall, the paper provides valuable insights into the sociolinguistic complexity of domestic life in a highly multilingual society, offering a fresh perspective on the intersection of language, gender, and family structures.

Is the work clearly and accurately presented and does it cite the current literature?

Yes

If applicable, is the statistical analysis and its interpretation appropriate?

Yes

Are all the source data underlying the results available to ensure full reproducibility?

Yes

Is the study design appropriate and is the work technically sound?

Yes

Are the conclusions drawn adequately supported by the results?

Yes

Are sufficient details of methods and analysis provided to allow replication by others?

Yes

Reviewer Expertise:

teaching language

We confirm that we have read this submission and believe that we have an appropriate level of expertise to confirm that it is of an acceptable scientific standard.
